# miRNA Profiling for Early Detection and Treatment of Duchenne Muscular Dystrophy

**DOI:** 10.3390/ijms20184638

**Published:** 2019-09-19

**Authors:** Heather C. Hrach, Marco Mangone

**Affiliations:** 1 Molecular and Cellular Biology Graduate Program, School of Life Sciences 427 East Tyler Mall, Tempe, AZ 85287 4501, USA; 2 Virginia G. Piper Center for Personalized Diagnostics, The Biodesign Institute at Arizona State University, 1001 S McAllister Ave, Tempe, AZ 85287, USA

**Keywords:** circulating miRNAs, Duchenne muscular dystrophy, biomarker

## Abstract

Duchenne muscular dystrophy (DMD) is an X-linked recessive genetic disorder caused by out of frame mutations in the dystrophin gene. The hallmark symptoms of the condition include progressive degeneration of skeletal muscle, cardiomyopathy, and respiratory dysfunction. The most recent advances in therapeutic strategies for the treatment of DMD involve exon skipping or administration of minidystrophin, but these strategies are not yet universally available, nor have they proven to be a definitive cure for all DMD patients. Early diagnosis and tracking of symptom progression of DMD usually relies on creatine kinase tests, evaluation of patient performance in various ambulatory assessments, and detection of dystrophin from muscle biopsies, which are invasive and painful for the patient. While the current research focuses primarily on restoring functional dystrophin, accurate and minimally invasive methods to detect and track both symptom progression and the success of early DMD treatments are not yet available. In recent years, several groups have identified miRNA signature changes in DMD tissue samples, and a number of promising studies consistently detected changes in circulating miRNAs in blood samples of DMD patients. These results could potentially lead to non-invasive detection methods, new molecular approaches to treating DMD symptoms, and new methods to monitor of the efficacy of the therapy. In this review, we focus on the role of circulating miRNAs in DMD and highlight their potential both as a biomarker in the early detection of disease and as a therapeutic target in the prevention and treatment of DMD symptoms.

## 1. Introduction

miRNAs are a class of short non-coding RNAs that function post-transcriptionally to regulate gene expression in a sequence-specific manner [1,2]. miRNA genes are located in clusters, transcribed with unique promoters, or found in intronic regions of protein-coding genes. Once transcribed and matured, they function through the assembly of a surrounding protein complex known as the RNA-induced silencing complex (RISC) [3]. Using the sequence of a given miRNA as a guide, the RISC is able to bind to a partially complementary region within the 3′untranslated region (3′UTR) of mature mRNAs, leading to silenced gene expression, either through translational repression or mRNA degradation [4].

miRNAs are involved in innumerous mammalian processes that are essential for development and survival [1]. miRNA activity plays a role in cellular proliferation and differentiation in all mammalian tissues. More specifically, several miRNA families have proven to be essential in the control of cardiac and skeletal muscle development [5]. Subsequent large scale experiments including serum profiling and microarray approaches have confirmed the relationship between miRNAs and typical muscle development, as several miRNA families are dysregulated in a variety of human neuromuscular diseases [6,7]. For example, changes in miRNA expression have been identified in patients with ALS, SMA, and myasthenia gravis, which are all diseases that lead to muscle dysfunction. In some cases, upregulation and knockdown experiments have proven successful in the treatment or improvement of symptoms in some of these conditions [7].

Duchenne muscular dystrophy (DMD) is currently the most commonly diagnosed form of muscular dystrophy and affects approximately 1 in every 3500 live male births. The condition is monogenic and is known to be caused by out of frame mutations in the dystrophin gene [8,9]. The dystrophin gene is the largest in the human genome, with its longest isoform spanning 79 exons and approximately 2.4 million base pairs [10]. Dystrophin forms a stabilizing connection between cytoskeletal actin and the transmembrane dystrophin-associated protein complex (DAPC), which absorbs mechanical stress after muscle contraction [11,12]. The absence of a functional dystrophin protein produces muscle inflammation, which, if left untreated, causes the dystrophic muscle to go through cycles of necrosis and muscle repair that are characterized by chronic inflammation. The muscle rebuilt during repair phases eventually becomes replaced with adipose and scar tissue, resulting in fibrosis. Ultimately this progressive muscle damage leads to a decline in function of the skeletal, cardiac, and respiratory muscles. 

Until recent years, the standard methods of diagnosis and symptom tracking included a serum creatine kinase test, sequence analysis, and evaluating patient scores in various ambulatory tests, including the North Star Ambulatory Assessment (NSAA), a group of standardized tests used to define motor ability, and the six-minute walk test [13]. Subsequent treatments focus on slowing down muscle deterioration with corticosteroids and managing symptoms of cardiomyopathy using ACE inhibitors and beta blockers. These medications, in combination with physical aids like braces, wheelchairs, and medical devices to assist respiratory function, are used to improve quality of life and extend overall lifespan [14]. 

Several decades of research have resulted in some experimental treatments that recently reached the clinical trial stage. These approaches aim to treat DMD on a molecular basis, and include exon skipping therapy and the delivery of a shorter version of dystrophin (minidystrophin) [15]. Exon skipping aims to restore the open reading frame of dystrophin by excluding the exon 51, which contains the most common out of frame mutations, resulting in a shorter but partially functional form of dystrophin [16]. The drug eteplirson, the morpholino antisense oligomer which facilitates this exon skipping event, has been recently granted accelerated FDA approval dependent upon upcoming studies that prove its efficacy [17,18]. The delivery of minidystrophin is typically achieved through the use of an adeno-associated virus (AAV) vector system [19]. The full-length dystrophin protein exceeds the packaging limit of this system, but the advent of this system has made delivering a shortened version of the protein a possibility. 

Both exon skipping therapy and minidystrophin delivery are very promising for treating DMD, but they are not yet routinely available to patients. One of the limitations of exon skipping therapy is that mutations in the dystrophin gene may occur in a different position from regions named mutational hotspots, which are generally located between exons 45 and 55. Exon skipping approaches currently aim to bypass mutations located within this region, and it has been estimated that 40% of DMD patients, which do not possess mutation between these exons, would not directly benefit from this treatment [20]. To overcome this obstacle, a recent study developed mutation-specific cocktails of antisense phosphorodiamidate morpholino oligomers targeting multiple exons [16]. This approach was successfully tested in human cell lines and murine models but has yet to reach the clinical trial phase.

The administration of minidystrophin has been met with its own unique set of limitations. The first attempt to use this system on human patients was attempted in 2006 and was met with results that were less promising than expected, and very limited expression of the minidystrophin cassette was observed in patients [21,22]. It has been hypothesized that this could be due to patient immune responses that suppress the desired expression of the new shortened version of the protein [21]. In the years following this study, extensive work has been done to improve the outcome of AAV-mediated delivery of minidystrophin [21,23,24,25]. There are several clinical trials that are currently attempting to deliver minidystrophin to human patients, although they are still in early phases and it is not yet clear if and when these will be available to all patients. Of note, while these approaches have clear translational value, their goal is to alleviate disease symptoms rather than to provide a complete cure for the disease.

While DMD treatment is the focus of intense research, there are currently very few methods to accurately detect the success of DMD treatment in young patients using non-invasive approaches. The damage of the skeletal, respiratory, and cardiac muscle is present before it is readily visible and the repair of this damage following various therapies can be slow and difficult to assess. Faster and more sensitive methods to measure the success of treatments like exon skipping and administration of minidystrophin could better facilitate the effectiveness of clinical treatment. This, in turn, could improve the quality of life for DMD patients, as more sensitive biomarkers will allow for a higher degree of accurate and personalized medicine throughout treatment. As a consequence of this need within the field of DMD diagnostics, the community has begun to evaluate circulating miRNAs as a tool that could prove useful both as a biomarker for treatment success and as a therapeutic target in the treatment of DMD. This area of research still has many open-ended questions and until the dystrophic muscle health can be definitively monitored and treated using non-invasive methods, it will remain a topic worthy of further focus and innovation. 

## 2. miRNA Signatures in Muscle and Muscle Disease

Myogenesis can be divided into three phases, as follows: embryonic, perinatal, and regenerative, the last of which occurs in response to muscle damage in mature muscle [26]. In regenerative myogenesis, the differentiation, maintenance, and repair of healthy muscle are tightly regulated processes that are controlled in part by miRNAs. Many known miRNAs are expressed in a tissue-specific manner [27,28], making them either present exclusively in the muscle or significantly enriched in this tissue. In particular, there are two families of miRNAs that include miR-1, miR-133a, miR-133b, and miR-206, which are essential in muscle development and maintenance and have thus been named myomiRs [5]. 

A recent study performed in a murine model of skeletal muscle injury revealed that administration of a mixture of some of the mature myomiRs (miR-1, miR-133, and miR-206) was able to accelerate the regeneration of muscle, prevent fibrosis at the site of injury, and increase the expression of classic markers of regenerative myogenesis, including myoD and Pax7 [29]. A second study, which analyzed muscle tissue samples from patients affected by 10 different muscular disorders, highlighted several trends in the dysregulation of miRNAs expression. Perhaps most interestingly, it was shown that between all 10 disorders included in the study, five miRNAs (miR-146b, miR-155, miR-214, miR-221, and miR-222) were consistently dysregulated in each sample, although they were alternatively either up- or downregulated depending on disease type [30]. These miRNAs have subsequently been named “dystromiRs.” They may be involved in cellular response to muscle damage and can potentially be exploited as highly specific biomarkers for each of these muscle disorders [31]. 

## 3. Circulating miRNAs as Biomarkers in the Early Detection of DMD

Circulating miRNAs have great potential to serve as biomarkers that are an accurate and minimally invasive alternative to muscle biopsies. Circulating miRNAs have already proven successful as informative biomarkers in other diseases [32,33,34] and are now the focus of several studies that aim at improving the field of DMD diagnostics (Table 1). For example, one such study has revealed that the levels of miR-1, miR-133, and miR-206 are inversely related to the NSAA score of DMD patients [35], meaning that as the severity of muscle damage worsens, the levels of these miRNAs in the blood increase (Table 1). It is important to note these results were consistent only with muscle function, and not with age, unlike CK tests, which frequently show fluctuations caused by age and previous steroid treatment, and are often unrelated to the severity of muscle damage [35]. Another recent study has shown that the upregulation of circulating miR-26a, miR-222, and miR-378a-5p can be used as a molecular signature for the presence of myocardial scars in DMD patients [36] (Table 1). 

A study in 2017, which focused on circulating miRNAs in DMD, demonstrated the utility of miRNAs not only as biomarkers for early detection, but also as a biomarker that is predictive of the severity of an individual patient’s case. Using digital PCR, this particular study revealed that miR-30c was present in higher levels in blood taken from DMD patients with comparatively better motor function (Table 1). This same study highlights the reliable nature of certain miRNAs in the detection of DMD, as they show that the miR-181a and miR-30c were consistently and significantly elevated at least 6-fold in the sera of both ambulatory DMD patients. Importantly, these results were consistent and without any association with the patient’s age or previous corticosteroid treatment [37] (Table 1). Finally, one study examined changes in miRNA levels in the urine of DMD patients and proposed an even more minimally invasive method of diagnosis [38]. This study finds that miR-29c-3p was significantly downregulated in the urine of DMD patients that were still considered ambulatory, and that miR-23b-3p and miR-21-5p were both downregulated in the urine of non-ambulatory DMD patients [38]. This work suggests that these could serve as accurate noninvasive biomarkers that are also sensitive enough to reflect the physical condition of the patient [38]. Taken together, these studies indicate that certain miRNAs appear to be consistently dysregulated in the context of DMD, and that changes in miRNA levels are detectable without performing analysis on tissue samples directly. The greater implications of these studies are that groups of miRNAs that are sensitive enough to detect DMD symptoms very early in disease progression may also be sensitive enough to detect subtle improvements in muscle health. This suggests that these dysregulated miRNAs may prove useful in the precise and non-invasive monitoring of patients that are undergoing various treatments for DMD. 

## 4. Circulating miRNAs as a Biomarker to Measure the Extent of Disease Progression in Muscle Tissues

In late-stage DMD progression, healthy muscle is progressively replaced with scar tissue, fat, and collagen, impairing muscle function. At this stage, significant variability in symptoms has been observed in patients and predicting the nature and extent of these muscle lesions is essential in order to manage individual patient outcomes. Finding biomarkers associated with the more progressive symptoms of DMD could mean earlier detection and more proactive treatment of some of these symptoms without performing repeated muscle biopsies on young patients. 

Several groups have recently started associating specific disease symptoms with altered miRNA levels in samples from DMD patients [39]. For example, one group has reported that the expression levels of miR-199a-5p, a miRNA which has been previously associated to fibrosis, were elevated in exosomes sourced from fibroblasts in DMD patients [39] (Table 1).

Progressive loss of cardiac function has also been linked to the progression of DMD. The ability to detect the extent of heart damage separately from damage occurring in the skeletal muscle using non-invasive approaches represents an ideal goal which has not yet been achieved. 

Recently miRNAs have also been shown as potential tissue-specific biomarkers for the effects of DMD on the function of cardiac muscle. Female carriers of dystrophin mutations do not present with severe paralysis like affected males, but often present with symptoms of cardiomyopathy as a result of uneven expression of wild-type and mutant versions of dystrophin throughout the muscle. The serum of female DMD carriers was found enriched with six miRNAs (miR-26a, miR-206, miR-222, miR-342, miR-378a-3p, and miR-378-5p) in blood samples taken from female carries of DMD with clinically confirmed cardiomyopathy (Table 1). In this particular study, two miRNAs, miR-29a and miR-144, were also found to be downregulated in the serum of female DMD carriers. Most importantly, miR-29c was significantly downregulated only in the female DMD carriers that showed functional and/or structural abnormalities of the cardiac muscle, and miR-29c downregulation was not detected in the serum of DMD carriers without symptoms of cardiomyopathy (Table 1). This work suggests that miR-29c can serve as an early biomarker for early stages of cardiomyopathies in DMD patients, even when biopsies do not yet necessarily show signs of the disease [40]. However, it remains to be seen whether or not the downregulation of this particular miRNA is also present in affected males and if this particular miRNA remains at wild-type levels prior to the onset of cardiomyopathy in males with DMD. This form of validation would make the tracking of miR-29c an even more promising method of following the tissue specific effects of dystrophin deficiencies. 

Finally, an in-depth analysis of expression patterns of dystromiRs in DMD patient samples has revealed that miR-1, miR-31, and miR-133b are significantly upregulated in patients that have been placed on a daily steroid regimen. As steroid use in DMD patients is understood to delay muscle deterioration, it can be inferred from this data that these miRNAs may be useful in evaluating the amount of remaining muscle mass in a DMD patient with more progressive symptoms. [41]

The specificity and reliability of the results detailed in these studies collectively suggest that miRNAs detected in serum could potentially serve as a robust tool to track the progression of symptoms for individual DMD patients, making personalized treatment plans more accurate and attainable. 

## 5. miRNAs as Therapeutic Targets in the Prevention of DMD Symptoms

In mouse models, the protein utrophin compensates for the absence of dystrophin. Utrophin is typically targeted by the miRNA let-7c. By blocking its binding site it is possible to rescue utrophin expression in the mouse model of DMD (*mdx*), which in turn improves muscle histology and reduces fibrosis [42]. It is important to note, however, that the compensatory utilization of utrophin is not seen naturally in human DMD patients, thus limiting the potential utility of this approach.

The progression of fibrosis in DMD patients also appears to be partially modulated by miRNA activity [37]. Based on an evaluation of samples from muscle biopsies taken from DMD patients, miR-21 is significantly elevated compared to healthy muscle tissues and its silencing reduces fibrosis in both *mdx* muscle and cultured fibroblasts taken from DMD patient biopsies [43] (Table 2). These results, in turn, suggest that both miR-21 and miR-29 families regulate overlapping biological processes in tissue fibrogenesis and, in principle, could be exploited to prevent extensive fibrosis in the early stages of DMD progression (Table 2). 

A notable symptom in both early and late stages of DMD is the increased susceptibility to muscle fatigue. This is in part due to the mislocalization of the neuronal form of the nitric oxide synthase enzyme (nNOS), following the deletion of its dystrophin binding sites within the rod region of the dystrophin protein [44]. A recent study of 617 miRNAs revealed that miR-34c and miR-708 were upregulated in DMD cells [45] (Table 2). Their validation experiments performed in human myoblasts showed that their inhibition results in increased nNOS expression, thus implicating these miRNAs as modulators of nNOS activity and potential therapeutic targets to combat premature muscle fatigue in DMD patients [45]. 

These works collectively suggest that miRNAs are intricately involved in muscle function and the up- or downregulation of miRNAs dysregulated in DMD could potentially be combined with current medications to improve the overall health of muscle affected by dystrophin deficiencies. 

## 6. miRNAs as Therapeutic Targets for the Treatment of Late-Stage Non-Ambulatory DMD 

While miRNAs have shown promise as biomarkers for detection of disease, there are also several lines of evidence suggesting a potential usage as a method of measuring success of disease treatment (Table 2). Previous studies have shown that the myomiRs, which are known to regulate myogenesis and are significantly dysregulated in DMD serum, can be restored to normal levels in *mdx* mice following exon skipping therapy, which ameliorates the DMD symptoms [46] (Table 2). This data suggests that monitoring the abundances of these three miRNAs in serum of DMD patients could potentially report treatment success. The effects of treatments like exon skipping, steroid treatment, and expression of minidystrophin may present slowly and subtly, meaning that monitoring myomiRs may be an ideal method to efficiently monitor the effectiveness of a given treatment approach. 

Fibrosis is a symptom of DMD that is pervasive, present in all patients, and is a major contributor to paralysis and loss of muscle function. For these reasons, many studies that aim to find therapeutic targets in the treatment of DMD focus on the pathways that control fibrosis. One of these studies has shown that treatment of myoblasts with synthetic miR-29 is able to slow down fibrosis and, in turn, promote muscle regeneration [47]. In addition to the aforementioned potential utility of miR-199a-5p as a marker of fibrosis, its expression could be, in principle, manipulated to combat fibrosis. A recent study performed on muscle-derived exosomes found that either transfection or delivery of exosomes loaded with miR-199a-5p induces a fibrotic response in normal fibroblasts. Furthermore, the injection of exosomes into healthy mouse muscle was also able to induce a fibrotic response that was increased in comparison to injection of control exosomes [39]. Another study that searched for targets that could be used to combat fibrosis found miR-675 and miR-21 to be involved in a signaling pathway that could potentially be exploited to reduce the effects on fibrosis in DMD muscle. It was shown in this work that the synthetic preimplantation factor, termed sPIF, was able to deplete collagen expression in *mdx* mice through the upregulation of miR-21 and miR-675. This work suggests that these two miRNAs could potentially be used in the treatment of either early or late stage fibrosis in cardiac, skeletal, or diaphragm muscle [48]. 

As previously mentioned, one of the most promising methods of treatment for DMD is the restoration of dystrophin expression. However, responses to exon skipping therapy have been faced with variable expression of dystrophin within individual patients and across groups of patients. It has been proposed that this could be caused by post-transcriptional regulation of restored dystrophin protein. In fact, one study has shown that there are three validated miRNAs (miR-146b/miR-374a/miR-31) that regulate dystrophin expression and that high levels of these miRNAs resulted in decreased success of dystrophin expression in exon skipping experiments [49]. This concept was also previously proposed in a study that found miR-31 inhibition can increase the amount of dystrophin protein expressed following rescue experiments designed to restore dystrophin expression [50]. Following further validation of these results, it appears that the inhibition of these miRNAs could potentially bolster the success of exon skipping therapies like etiplerson [49].

The current standard of treatment in DMD patients with advanced symptoms primarily relies on medical devices that facilitate movement and respiration and medications that manage the discomfort associated with severe muscle deterioration. The use of miRNA therapeutics in non-ambulatory patients has the potential to improve the overall condition of damaged muscle and in turn improve quality of life for DMD patients with significant fibrosis. 

## 7. Conclusions and Future Directions

In conclusion, miRNAs show promise both as a biomarker to improve the diagnosis and monitoring of DMD progression and treatment, and as therapeutic targets that can be up- or down-regulated to ameliorate both early and late stage symptoms of DMD. There is an outstanding need for a highly sensitive and minimally invasive method to track symptom progression and whether or not a particular treatment is successful in improving these symptoms. The studies summarized here suggest that the monitoring of miRNAs may directly address this need. It remains to be determined whether or not there are individual miRNAs or classes of miRNAs that can be consistently detected in serum and have the ability to indicate whether or not a patient has DMD, or another form of muscular dystrophy prior to the onset of loss of ambulation. It is also important to note that because many of the miRNAs described in this review are implicated in widespread muscle function, future diagnostic methods may need to depend on groups of miRNAs, rather than individual miRNAs, when searching for a robust method to detect symptoms of DMD. A number of comorbidities are associated with DMD, including but not limited to dysphagia, behavioral and cognitive conditions, and high or low body weight. It is possible that these variable factors could independently influence miRNA expression at different rates between patients, making it more plausible to examine groups of miRNAs together as a diagnostic tool.

There are several outstanding questions regarding the ability of miRNAs to act as biomarkers that are accurate at the tissue specific level, especially in the context of muscle deterioration in the diaphragm. As respiratory dysfunction can be considered one of the lethal symptoms of DMD, further work identifying miRNAs that can predict the onset of respiratory dysfunction is still needed. Additionally, more studies are needed to confirm the results of combining miRNA manipulation with drugs such as etiplerson to validate their role in the variable expression of dystrophin following restoration of the dystrophin open reading frame. 

Finally, the circumstances under which miRNAs can and should be administered therapeutically requires careful consideration. The progression of DMD symptoms is not identical from one patient to the next. This fact, combined with the fact that miRNA expression and function is tissue specific, means that miRNAs may carry out different functions in each tissue and may not be an appropriate treatment option under all circumstances. miRNAs with potentially opposing roles in different tissues should be further examined before considering them as therapeutic targets in the treatment of some tissue specific symptoms seen in specific cases. Ideally, future studies will strive to combine miRNA detection, administration, or inhibition with methods like exon skipping to improve diagnosis and symptom management. 

## Figures and Tables

**Table 1:** Summary of circulating miRNAs found dysregulated in the serum of DMD patients and female carriers with out of frame dystrophin mutations.

miRNA	Description	Reference
miR-1	Upregulated in the serum of DMD patients	[35]
miR-31		[41]
miR-133		
miR-206		
miR-222	Upregulated along with the occurrence of myocardial scars	[36]
miR-26a		
miR-378a-5p		
miR-181	Correlates positively with motor function in DMD patients	[37]
miR-30c		
miR-29c-3p	Downregulated in the urine of DMD patients	[38]
miR-23b-3p		
miR-21-5p		
miR-199a-5p	Elevated in exosomes sourced from DMD muscle	[39]
miR-26a	Upregulated in female carriers with cardiomyopathy	[40]
miR-206		
miR-22		
miR-342		
miR-378a-3p		
miR-378-5p		
miR-29c	Downregulated in female carriers with cardiomyopathy	[40]
miR-144		

**Table 2:** Summary of miRNAs that can be potentially manipulated as therapeutic targets in the treatment of DMD.

miRNA	Description	Reference
miR-675	sPIF mediated reduction of muscle fibrosis via upregulation of miR-675	[48]
miR-21	Reduction of miR-21 expression reduces fibrosis	[48]
		[43]
miR-31	Inhibition increases dystrophin rescue	[49]
miR-146b		[50]
miR-374a		
miR-29	Treatment with synthetic miR-29 slows fibrosis and promotes muscle regeneration	[47]
miR-708	Inhibition results in increased nNOs expression	[45]
miR-34c		
miR-199a-5p	Overexpression induces fibrotic response in *wt* fibroblasts and mouse models	[39]
